# J2R/F4L deleted oncolytic vaccinia virus synergizes with tumor specific killer (ELANE) promotes antitumor immunity with superior safety

**DOI:** 10.3389/fimmu.2026.1861346

**Published:** 2026-07-21

**Authors:** Yu Huang, Liming Wang, Shan Mei, Fei Zhao, Lingwa Wang, Yu Xie, Zhao Gao, Jiaxun Wang, Ziyue Kong, Yurong He, Yueyue Shi, Wenkai Guo, Bin Ai, Jugao Fang, Fengwen Xu, Fei Guo

**Affiliations:** 1 Key Laboratory of Pathogen Infection Prevention and Control (Ministry of Education), State Key Laboratory of Respiratory Health and Multimorbidity, National Institute of Pathogen Biology, Chinese Academy of Medical Sciences & Peking Union Medical College, Beijing, China; 2 National Institute of Pathogen Biology and Center for AIDS Research, Chinese Academy of Medical Sciences & Peking Union Medical College, Beijing, China; 3 Department of Otorhinolaryngology, Head and Neck Surgery, Beijing Tong Ren Hospital, Capital Medical University, Beijing, China; 4 Department of Neurosurgery, The First Affiliated Hospital of Xi’an Jiaotong University, Xi’an, Shaanxi, China; 5 Department of Medical Oncology, Beijing Hospital, National Center of Gerontology, Institute of Geriatric Medicine, Chinese Academy of Medical Sciences, Beijing, China

**Keywords:** human neutrophil elastase, immunogenic cell death, oncolytic virus, tumor microenvironment, vaccinia virus

## Abstract

**Background:**

Oncolytic virotherapy (OVT) represents a promising approach for cancer treatment, employing oncolytic viruses (OVs) that selectively infect and lyse tumor cells while promoting an antitumor immune microenvironment. Vaccinia virus (VACV) serves as an attractive oncolytic vector due to its favorable safety profile, ease of genetic modification, and inherent tumor selectivity.

**Methods:**

To enhance both safety and tumor-targeting capability, we constructed a recombinant vaccinia virus, VV-dTF/EE, by deleting the viral *J2R* and *F4L* genes and inserting the human neutrophil elastase (ELANE) gene, which exhibits tumor-killing activity. Mechanistic studies evaluated with virus replication selectivity, cell apoptosis, genomic damage, immunogenic cell death, alongside analysis of the immune microenvironment. Efficacy was tested in multiple tumor cell models *in vitro* and lung cancer models *in vivo*.

**Results:**

In tumor cell lines and mouse tumor models, VV-dTF/EE demonstrated tumor-restricted replication, potent oncolytic effects, and induction of immunogenic cell death. Furthermore, VV-dTF/EE augmented VACV-induced antitumor immunity by increasing CD8^+^ T cell infiltration and suppressing M2-like macrophage polarization.

**Conclusions:**

This VV-dTF/EE revealed tumor-selective replication and killing ability while modifying the tumor immune microenvironment to elicit immunogenic cell death. Our findings highlight a novel strategy for safe and effective tumor immunotherapy through dual-gene deletion and ELANE expression in an oncolytic vaccinia platform.

## 1 Introduction

Broad-spectrum anticancer therapies are challenging due to the spatial and temporal tumor heterogeneity (1). Owing to the broad therapeutic efficacy and specificity, innate immunity holds great potential for the development of pan-cancer therapies (2). Neutrophils, also known as polymorphonuclear cells (PMNs), is the most prevalent immune cells that act as crucial effectors of innate immunity (3). Serum-free conditioned media from human PMNs displayed tumor-selective cytotoxicity, eliminating 35 cancer cell lines spanning 11 tumor types without harming normal cells (4). Functional studies revealed that human neutrophil elastase (ELANE), a serine protease expressed in neutrophils, is the major protein driving this selective anticancer effect (4). Single nucleotide polymorphisms in *ELANE* have been associated with periodic neutropenia and severe congenital neutropenia (5–7). It has also been shown to increase human leukocyte antigen (HLA) class I expression in breast cancer cells (8), and promoting tumor susceptibility to cytotoxic T lymphocyte (CTL) (9). ELANE liberates the CD95 death domain (DD) that interacts with cancer cell specific histone H1 isoforms (4). Furthermore, it triggers mitochondrial translocation of histone H1, which is elevated in cancer cells compared to non-cancer cells (10, 11), and inhibits distant metastasis by inducing a CD8^+^ T cell-mediated abscopal effect (4). Therefore, ELANE holds a great potential as a pan-cancer therapeutic. However, abundant serine protease inhibitors (serpins) in blood and tumor microenvironment constitute a major barrier to ELANE’s anti-tumor efficacy (12). A delivery system is thus needed to deliver ELANE intratumorally, avoiding the inhibition by serpins.

Oncolytic viruses (OVs) have become an emerging class of cancer therapeutics (13, 14). They can selectively replicate in tumor cells, enhance local antitumor immunity, deliver tumor killing genes such as ELANE (15–17). Diverse viruses have oncolytic property, including DNA viruses (e.g., adenovirus, vaccinia virus (VACV), herpes virus) and RNA viruses (e.g., reovirus, measles virus, New Castle disease virus (NDV), vesicular stomatitis virus (VSV)). DNA oncolytic viruses often offer a few advantages such as their large genome size, high genetic stability, and robust replication capacity (15). For example, VACV has a genome of ~190 kb, allows the insertion of long exogenous therapeutic genes (18). VACV replicates in the cytoplasm, thus does not integrate into host DNA (19). VACV has been widely used as smallpox vaccine, has a proven safety profile (20). Compared to other DNA viruses such as adenoviruses, VACV has inherent tumor tropism (21), which could be further engineered to improve its tumor selectivity by deleting genes such as thymidine kinase (*J2R*) (22, 23)and small subunits of heterodimeric ribonucleoside diphosphate reductase (RNR) complex (*F4L*) (24). The engineered VACV is able to replicate through the use of high nucleic acid metabolism in tumor cells, thus presents higher selective killing of cancer cells.

In addition to directly killing tumor cells, VACV also activates local anti-tumor immunity (25). This is because tumor cell lysis results in the release of damage-associated molecular patterns (DAMPs) and tumor-associated antigens (TAAs) which elicit anti-tumor immune responses. This type of cell death is also called immunogenic cell death (ICD). The released DAMPs can include high mobility group box 1 (HMGB1), adenosine triphosphate (ATP) and surface expression of calreticulin (CRT) (26, 27). These DAMPs promote the maturation of dendritic cells which present the TAA to T cells, activate tumor-specific adaptive immunity, and impact the tumor microenvironment (TME) (28).

In cancer immunotherapy, the immunological property of TME often determines disease prognosis and therapeutic success (29). In this study, we have engineered a VACV by inserting the tumor-selective cytotoxic gene *ELANE* into the thymidine kinase (*J2R*) locus, while simultaneously deleting the *F4L* gene encoding ribonucleotide reductase to attenuate viral nucleotide metabolism. This dual-modification strategy combines tumor-selective viral replication with tumor-specific killing by ELANE, thus both ensuring safety and increasing oncolytic efficacy. The recombinant VACV induces robust ICD through viral infection and ELANE expression in cancer cells, which drives potent cytotoxic T lymphocyte (CTL) differentiation and effector T-cell expansion. At the same time, this VACV alleviates immunosuppressive barriers, including T-cell exhaustion and tumor-associated macrophage (TAM), thereby promoting systemic antitumor immune responses. As a result, this approach ensures safety while fostering a TME conducive to the recruitment and activation of anti-tumor immune cells, thereby enhancing tumor cell eradication.

## 2 Materials and methods

### 2.1 Construction of recombinant VACV

Human neutrophil elastase (ELANE) and porcine pancreatic elastase (PPE) were inserted into *J2R* locus of the VACV Tiantan strain through homologous recombinant with *J2R* upstream and downstream sequences as previously described (30). After five rounds of plaque purification, successful insertion of ELANE and PPE into the viral genome was confirmed by PCR and DNA sequencing. Purified recombinant VACVs were amplified by infecting Vero cells, their titers were determined by plaque assay. Primers used were as follows:

J1RJ3RF: 5’-CGATGTTCTTCGCAGATGATG-3’.

J1RJ3RR: 5’-CTTTGTTACGGACGATCTTATTAAGGTAGT-3’.

The dTF and dTF/EE VACVs were generated by recombination with donor plasmid using *F4L* upstream and downstream homologous sequence. Primers used were as follows:

F4LF: 5’-ATTGCCTCCTATGGCATCAAT-3’.

F4LR: 5’-GAACCCATCCTTGCACCAAAT-3’.

### 2.2 Cell lines

The human liver cancer cell line Huh7.5.1 cells were purchased from Shanghai Biological Technology (Shanghai, China), together with human normal liver cell line THLE-2 (ATCC, CRL-2706) and human bladder cancer cell line 5637 (ATCC, HTB-9), were maintained in RPMI 1640 medium. Human lung cancer cell line A549 (ATCC, CCL185), human embryonic lung fibroblast MRC5 (ATCC, CCL-171) and mouse lung cancer cell line LLC (ATCC, CRL-1642) were cultured with DMEM medium. All cell lines were supplemented with 10% fetal bovine serum (FBS) and 100 U/ml penicillin-streptomycin. Cells were maintained in a humidified incubator at 37 °C with 5% CO_2_.

### 2.3 Virus proliferation and cytotoxicity assays

The ability of viruses to replicate in cultured cells was determined by plaque assays in Vero cells. Cells were seeded in 12-well plates (2×10^5/well) and infected at an MOI of 0.005. Following a 2 hours adsorption period at 37 °C, the inoculum was removed, and cells were washed twice with phosphate-buffered saline (PBS), then overlaid with culture medium. Infected cells were harvested at 0, 12, 24, 36, and 48 h post-infection. At each time point, cell lysates were subjected to three successive freeze-thaw cycles (freezing at −80 °C and thawing at room temperature) to release intracellular virions. The lysates were then centrifuged at 1000×g for 10 min at 4 °C to remove cell debris, and the resulting supernatants were collected as virus stock solutions. Virus titers were determined by standard plaque assay on Vero cell as described previously (31). Briefly, ten−fold serial dilutions of the supernatants were added to confluent Vero cells in 12−well plates. Cells were then covered with semi−solid medium, and incubated for 3 days. Plaques were visualized by staining with crystal violet, and titers were calculated as plaque-forming units per milliliter (PFU/mL).

CCK8 (Cell Counting Kit 8) was used to measure cell survival following virus infection. Briefly, cells were infected in 96-well plates (2×10^4/well) at indicated MOIs, followed by adsorption, and replacement with low-serum culture medium (1% FBS). After 72 hours of cultivation, 10μl CCK8 (MCE, HY-K0301) reagent was added, after an incubation at 37 °C for 1 hour, absorbance at 450nm was measured using a microplate reader. Cell survival was calculated in reference to mock-infected wells.

### 2.4 Expression and protease activity of ELANE secreted from recombinant virus-infected cells

Vero cells were infected with the virus at an MOI of 1, and the supernatant was collected 24 h post-infection. The supernatant was subjected to ultrafiltration centrifugation using a 50 kDa MWCO (4 °C, 4000×g, 30 min), and the resulting filtrate (flow-through) was collected. ELANE expression in the filtrate was assessed by immunoblotting. After confirming the absence of infectious virus particles in the filtrate by plaque assay, 5637 bladder cancer cells were incubated with the virus-free filtrate for 24 h. Finally, the cancer cells were harvested and processed for Western blotting to detect CD95 (Fas) cleavage.

### 2.5 Western blot analysis

Cells were infected with recombinant VACVs for 24 h or 48 h. Post infection, cells were harvested and lysed in RIPA buffer. The amount of protein was determined with the BCA Protein Assay Kit (ThermoFisher, A55860). Cell extracts of equal protein amounts were mixed with SDS loading buffer, resolved in SDS-PAGE, and transferred to the NC membrane. Membranes were blocked in 1xPBS containing 5% BSA at room temperature for 1 h before incubation with primary antibodies against PARP (CST, 9542S), Caspase3 (CST, 9662S), GSDME (Proteintech, 13075-1-AP), γH2AX (Sigma, 05-636), ELANE (GeneTeX, GTX113175), CD95 (Proteintech, 13098-1-AP), or actin (Sigma, A1978). HMGB1 (CST, 6893S) and HSP90 (Proteintech, 13171-1-AP) were detected in culture supernatants of infected cells. The IRDye secondary antibodies (1:20,000; LI-COR Biotechnology) was used following primary antibody. Protein bands were visualized on the LI-COR Odyssey instrument (LI-COR Biotechnology). The specific information regarding the use of all antibodies is provided in **Table 1** of the **Supplementary Materials**.

**Table 1:** Antibody information.

Antibody name	Target	Fluorophore	Source	Identifier	Dilution ratio
PARP Antibody	PARP	–	CST	9542S	1:1000
Caspase3 Antibody	Caspase3	–	CST	9662S	1:1000
HMGB1 (D3E5) Rabbit Monoclonal Antibody	HMGB1	–	CST	6893S	1:1000
HSP90 Polyclonal antibody	HSP90	–	Proteintech	13171-1-AP	1:1000
DFNA5/GSDME Polyclonal antibody	GSDME	–	Proteintech	13075-1-AP	1:1000
Fas/CD95 Polyclonal antibody	CD95	–	Proteintech	13098-1-AP	1:1000
Anti-β-Actin (ACTB) Antibody	Actin	–	Sigma	A1978	1:1000
Anti-phospho-Histone H2A.X (Ser139) Antibody, clone JBW301	γH2AX	–	Sigma	05-636	1:1000
Neutrophil elastase Antibody [N2C3]	ELANE	–	GeneTeX	GTX113175	1:1000
allophycocyanin (APC)-labeled anti-CRT polyclonal antibody	calreticulin	APC	Proteintech	10427-2-AP	1:1000
BV786 Rat Anti-Mouse CD45	Mouse CD45	BV786	BD Bioscience	564225	1:200
PE-Cy™7 Hamster Anti-Mouse CD3e	Mouse CD3	PE-Cy™7	BD Bioscience	552774	1:200
RB705 Rat Anti-Mouse CD4	Mouse CD4	RB705	BD Bioscience	570258	1:200
BV510 Rat Anti-Mouse CD8a	Mouse CD8	BV510	BD Bioscience	563068	1:200
BB515 Rat Anti-Mouse CD25	Mouse CD25	BB515	BD Bioscience	564424	1:100
PE Rat Anti-Mouse IFN-γ	Mouse IFN-γ	PE	BD Bioscience	554412	1:100
PE-CF594 Hamster Anti-Mouse CD279 (PD-1)	Mouse CD279 (PD-1)	PE-CF594	BD Bioscience	562523	1:100
RB705 Rat Anti- Mouse CD11b	Mouse CD11b	RB705	BD Bioscience	570292	1:200
BV421 Rat Anti-Mouse F4/80	Mouse F4/80	BV421	BD Bioscience	565411	1:100
BV650 Rat Anti-Mouse CD86	Mouse CD86	BV650	BD Bioscience	564200	1:100
PE Rat Anti-Mouse CD206	Mouse CD206	PE	BD Bioscience	568273	1:100
BV711™ anti-mouse CD366 (Tim-3)	mouse CD366 (Tim-3)	BV711™	Biolegend	119727	1:50
BV421™ anti-mouse FOXP3	mouse FOXP3	BV421™	Biolegend	126419	1:50

### 2.6 Quantification of secreted ATP

Cells (2×10^4) were infected with indicated recombinant VACV at the indicated MOI in a 96-well plate. Medium from each well was collected 24 h post-infection, levels of ATP were determined using luminescence-based ATP determination kit (MCE, HY-K0314) with CellTiter-Glo Luminescent Cell Viability Assay (Promega).

### 2.7 CRT exposure assay

Cells (5×10^5^) were plated into six-well plates and infected with indicated recombinant VACV. After infection, cells were harvested, washed twice with 1×PBS, and incubated with allophycocyanin (APC)-labeled anti-CRT polyclonal antibody (Proteintech, 10427-2-AP) at room temperature for 15 min. Finally, the CRT exposure was detected by flow cytometry.

### 2.8 Mouse study

All experiments were done following guidelines and approved by BeiJing Huilin Zegu Biotechnology Co., LTD. Institutional Animal Care and Use Committee (Approval No. HLZG-DWLL-2024-1209-08).

For the xenograft model, 4–6 weeks athymic female Balb/c mice were acclimatized for 1 week and injected with 1 × 10^7^ A549 cells subcutaneously on right side of the flank. For syngeneic model, 4–6 weeks old C57BL/6 mice were acclimatized and injected with 1 × 10^6^ LLC cells subcutaneously into right lower flank. When tumors reached an approximate volume of 150 mm^3^ (A549) or 100 mm^3^ (LLC), mice were sorted into treatment groups. Mice were either injected with PBS or the indicated virus. Weight and tumor volumes were measured once every three days. Tumor volume (mm^3^) was calculated as (lengthxwidth2)/2. In compliance with ethical guidelines, tumor size was restricted to ≤ 2500 mm^3^. Mice were monitored every three days (tumor size, body weight, survival status and adverse reaction) and euthanized via intraperitoneal injection of Zoletil 50 (50 mg/kg) and cervical dislocation if tumors exceeded this limit or if body weight loss exceeded 20%.

To assess bio-distribution of viruses, mice treated as above were euthanized on day 4 after treatment for A549 model. Tumors and organs were harvested and homogenized, virus titers were determined by plaque assay and VACV genome PCR target on E9L fragment (32), the primers were used as followed:

E9L-F: 5’-TGGCAAACCGTAACATACCG-3’.

E9L-R: 5’-AGGCCATCTATGATTCCATGC-3’.

### 2.9 Tumor immune cell analyses

Tumor tissues from mice were weighed, minced into small pieces and digested with the Miltenyi Biotec mouse tumor digestion kit (130-096-730). The tissues were gently homogenized and enzymatically digested to obtain a single-cell suspension. The cell suspension was filtered through a 70 µm cell strainer, centrifuged, and then treated with red blood cell lysis buffer for 5 minutes in the dark, followed by two washes with PBS. The harvested cells were first stained with Fixable Viability Stain 780 to discriminate live/dead cells. After washing and centrifugation, surface antigen staining was performed using antibodies against cell surface markers for 30 minutes at 4°C in the dark. The cells were washed and then subjected to fixation & permeabilization. Intracellular staining was subsequently performed using anti-FoxP3 and anti-IFNγ antibody (incubated for 30 minutes at 4°C). After two additional washes, the cells were acquired on a flow cytometer. The specific information regarding the use of all antibodies is provided in **Table 1** of the **Supplementary Materials**.

### 2.10 Immunohistochemical and immunofluorescent analysis of mouse tumor

Tumors were isolated on day 6, fixed in 4% paraformaldehyde for 24 h, embedded in paraffin blocks, and sectioned (in 5 mm thickness). To measure the ICD marker by immunohistochemistry, slides were stained with HSP90 and HMGBI. To analyze cytotoxic T cell by immunofluorescence, slides were stained with CD8 antibody and fluorescent secondary antibodies. Images were obtained with Pannoramic SCAN II and analyzed using Slide Viewer software.

### 2.11 Statistical analysis

Analysis of variance (ANOVA) was used when comparing multiple groups followed by Tukey’s multiple comparison test. Data for animal survival curves were analyzed by log-rank test. GraphPad Prism 10 software was used for statistical analysis. Data are expressed as variance ± standard error of the mean or standard deviation (SD). The numbers of animals included in each figure are indicated at the end of each legend. P-values are indicated within each figure.

## 3 Results

### 3.1 Replication and cancer cell killing properties of the VV-dTK/EE and VV-dTK/PE recombinant VACVs

**Figure 1:**
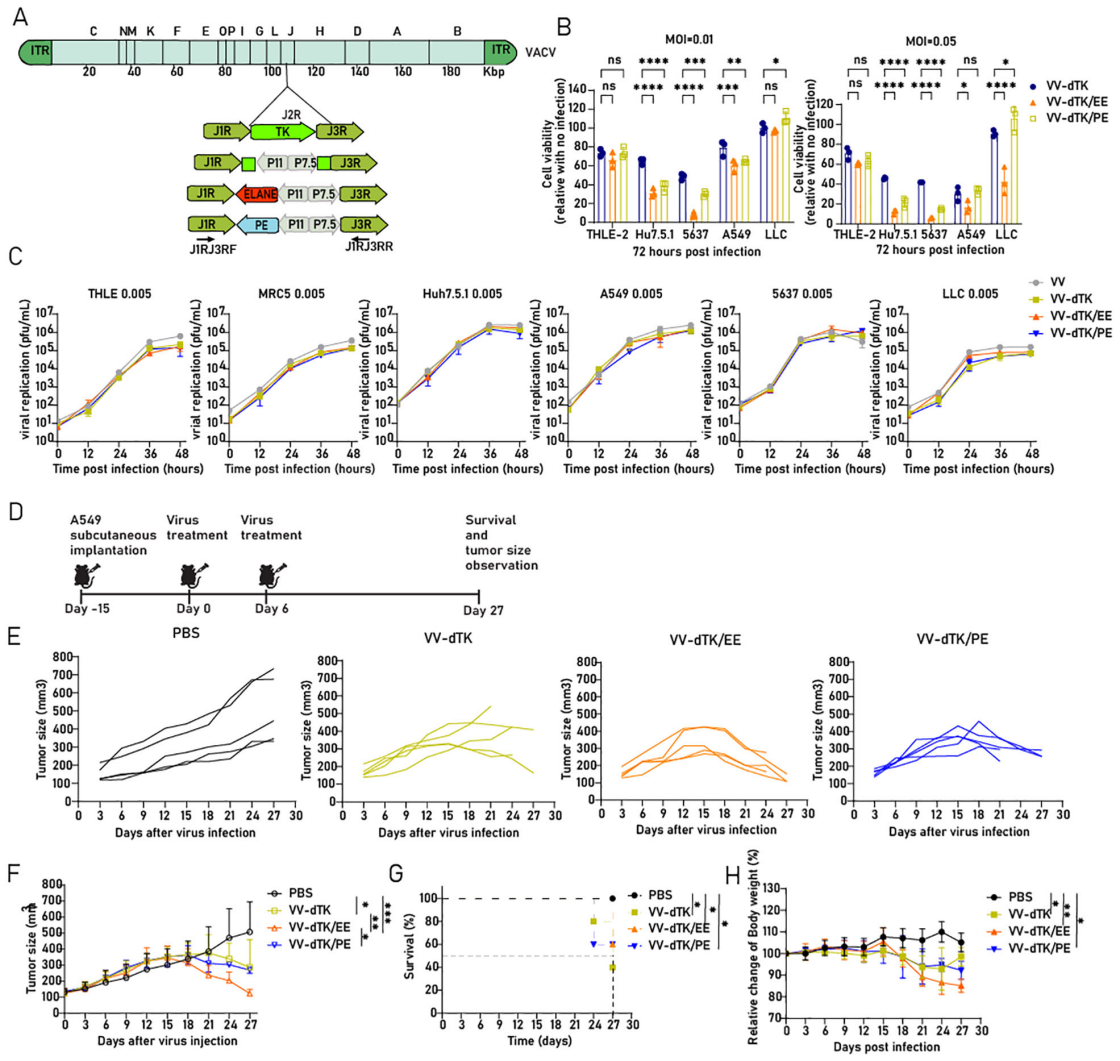
Replication and cancer cell killing properties of VV-dTK/EE and VV-dTK/PE. **(A)** Schematic representation of VACV recombinants VV-dTK/EE and VV-dTK/PE. Locations of PCR primers are denoted. The ELANE and PPE genes were inserted into thymidine kinase (TK) locus of VACV Tiantan strain by homologous recombination under the control of the early/late promoter p11. **(B)** Cell survival following infection with the indicated VACV recombinants. Cells were infected at the indicated MOI and grown with 1% serum. Uninfected cells were used as control. The statistical analysis was estimated by one-way ANOVA with Tukey’s correction. All data are presented as mean values ± SD. **(C)** Growth curves of the indicated VACV strains in human liver cell line THLE-2, human embryonic lung fibroblast MRC5, human liver cancer line Huh7.5.1, human bladder cancer cell line 5637, human non-small cell lung carcinoma line A549, and mouse lung tumor cell line LLC. Cells were infected at MOI = 0.005. Data are presented as mean values ± SD. **(D)** Schedule of A549 tumor implantation and VACV treatment. Balb/c nude mice harboring subcutaneous A549 tumors were randomized and injected twice (days 0 and 6) with an intratumoral dose of 5x10^5^ PFU of VACV. PBS was used as the control. **(E–H)** Tumor size in individual animals **(E)**, mean tumor size after treatments **(F)**, overall survival **(G)** and body weight changes **(H)** are plotted (n=5). The statistical analysis was estimated by Two-way ANOVA with Tukey’s correction for the data shown in **(F)** and **(H)**. Log-rank test was performed for the data in **(G)**. All data are presented as mean values ± SD. *p < 0.05; **p < 0.01; ***p < 0.001; ****p < 0.0001; ns, not significant.

Using homologous recombination, we inserted the human neutrophil elastase ELANE or porcine pancreatic elastase (PPE) (which could also have the anti-tumor activity) sequences into the *J2R* locus in the genome of VACV Tiantan strain under the control of late promoter p11 (**Figure 1A**). *J2R* encodes the viral thymidine kinase, a critical enzyme in the salvage pathway for nucleotide biosynthesis (33). The recombinant VACVs were named VV-dTK/EE and VV-dTK/PE. They were identified by PCR using primers targeting homologous arm sequence (**Supplementary Figure 1A**), and further purified with plaque selection. Expression of ELANE or PPE was detected in the infected Vero cells. Due to the absence of a commercially available antibody against PPE, expression of ELANE or PPE was detected using an anti-ELANE antibody. A faint band was also observed in the PPE lane, likely attributable to cross-reactivity of the antibody (**Supplementary Figure 1B**). To detect protease activity of ELANE in supernatants of recombinant virus infected Vero cells, the supernatant was collected and subjected to 50 kDa MWCO ultra centrifugal filters. ELANE expression was detected in the filtrate, indicating that ELANE was released into the supernatant and passed through the 50−kDa cutoff filter. After confirming the absence of infectious viral particles in the filtrate through plaque assay, 5637 bladder cancer cells were incubated with the virus−free filtrate for 24 h. Western blot analysis revealed a distinct cleaved fragment of CD95 in addition to the full−length CD95 band, demonstrating that secreted ELANE induced CD95 cleavage in tumor cells (**Supplementary Figure 1C**).

The effect of recombinant VACV on cell survival was determined using CCK8-based viability assay. Under low-serum condition (1% FBS), VV-dTK killed cancer cells more significantly than killing normal THLE-2 hepatocytes. To be more specific, 74.5% of THLE-2 cells were viable at MOI 0.01 and 71.5% at MOI 0.05 after 72 hours of infection; viability of Huh7.5.1, 5637, and A549 cells dropped to 64.6%, 48.4%, and 78.5% at 72 h post infection (hpi). Strikingly, VV-dTK/EE demonstrated even greater cytotoxicity compared to VV-dTK in human cancer cells, by reducing cell viability to 31.1%, 7.9%, and 60.2% at 72 hpi with MOI of 0.01 in Huh7.5.1, 5637, and A549 cells. Although LLC cells did not well support VACV replication, they were subject to killing by VV-dTK/EE much more than by VV-dTK at 72 hpi at MOI = 0.05. In contrast, VV-dTK/PE caused moderate killing of cancer cells compared to VV-dTK/EE (**Figure 1B**).

To characterize the replication of the recombinant VACVs, VV-dTK/EE and VV-dTK/PE were used to infect human liver cells THLE-2, human embryonic lung fibroblast MRC5, human liver cancer cell line Huh7.5.1, human bladder cancer cell line 5637, human non-small cell lung carcinoma A549 cells, and mouse lung tumor cell line LLC. Both VV-dTK/EE and VV-dTK/PE replicated more efficiently in cancer cells than in normal cells. With low serum (1%), both recombinant VACVs produced much higher virus titers (in the range of 10^6^ PFU/mL) in cancer cells than in human normal liver cells THLE-2 and human embryonic lung fibroblast MRC5 (about 1x10^5^ PFU/mL). VV-dTK/EE and VV-dTK/PE replicated less well in mouse lung cancer cell LLC (**Figure 1C**), which is in agreement with other studies showing that mouse cancer cells are less supportive of VACV infection (34, 35).

Collectively, these data suggest that the recombinant VACV VV-dTK/EE and VV-dTK/PE replicated efficiently in cancer cells and VV-dTK/EE exhibited significantly stronger oncolytic effects than VV-dTK and VV-dTK/PE.

### 3.2 VV-dTK/EE induces tumor cell apoptosis and immunogenic cell death

To understand how VACV-dTK/EE selectively kills cancer cells, we measured apoptosis in cancer and non-cancer cells after exposure to this recombinant VACV. Compared to VV-dTK that induced moderate apoptosis in cancer cells, VV-dTK/EE infection led to more severe apoptosis as shown by more cleavage of caspase-3 and PARP, and stronger DNA damage which was measured by the phosphorylation of H2AX (**Supplementary Figure 1D**). During VV-dTK/PE infection, the apoptotic and DNA damage signaling induced in 5637 cells was comparable to that of VV-dTK/EE. In Huh7.5.1 cells, the effect was slightly weaker than VV-dTK/EE but stronger than the VV-dTK group (**Supplementary Figure 1D**). In contrast, no enhancement of apoptotic signaling or genomic damage was observed in A549 and LLC cells with PE expression compared to VV-dTK. In addition, VV-dTK/EE infection also led to significant cleavage of pyroptosis effector GSDME, a substrate of caspase-3 (**Supplementary Figure 1D**), which indicates that VV-dTK/EE infection also causes pyroptotic cell death in addition to apoptosis, together contributing to its considerable antitumor effect. The cleavage and activation of GSDME triggered by VV-dTK/PE infection were also reduced in Huh7.5.1, A549, and LLC cells relative to VV-dTK/EE. ELANE kills tumor cells by proteolytically liberates the CD95 death domain. Surprisingly, VV-dTK infection markedly upregulated CD95 expression in cancer cells, which provides more CD95 for ELANE to cleave, together enhancing tumor killing by VV-dTK-EE (**Supplementary Figure 1D**).

In addition to apoptosis and pyroptosis, VACV can kill cancer cells by inducing immunogenic cell death (ICD). Our data showed that VV-dTK induced moderate ICD, whereas VV-dTK/EE infection further increased the release of ICD makers HMGB1, HSP90 (**Supplementary Figure 2A**) and ATP (**Supplementary Figure 2B**) in Huh7.5.1, 5637, A549 and LLC cell culture supernatants. ICD is also characterized by the translocation of calreticulin (CRT) from the cytoplasm to the plasma membrane, which can be detected by flow cytometry. Strikingly, VV-dTK/EE infection substantially elevated the levels of CRT on the plasma membrane (**Supplementary Figure 2C**). However, infection by VV-dTK/PE in tumor cells (A549 and LLC) resulted in reduced release of HSP90 and HMGB1 compared to VV-dTK/EE. Similarly, ATP secretion and CRT translocation to the cell membrane were also attenuated in VV-dTK/PE-infected tumor cells (Huh7.5.1, 5637, A549 and LLC) relative to the VV-dTK/EE group. Together, these data demonstrate that VV-dTK/EE further potentiates ICD of cancer cells compared with VV-dTK and VV-dTK/PE.

Next, we further assessed tumor cell killing by VV-dTK/EE in the xenograft models using human non-small cell lung cancer (NSCLC) A549 cells in Balb/c nude mice (**Figure 1D**). Two intra-tumoral injections of VV-dTK with a 6-day interval induced significant tumor regression, VV-dTK/EE exhibited a greater inhibition of tumor growth than VV-dTK and VV-dTK/PE (**Figures 1E, F**). However, we observed treatment-related adverse effects in the VACV infection groups, such as body weight loss, skin coarsening, and mortality (**Figures 1G, H**). These observations underscore the need for additional viral engineering to increase tumor selectivity and improve safety.

Taken together, based on the combined assessment of cellular viability, apoptosis induction, genomic damage, GSDME cleavage activation, ICD signaling (HSP90/HMGB1 release, ATP secretion, and CRT exposure), and antitumor efficacy in A549 xenograft model, VV-dTK/EE consistently demonstrated superior therapeutic potential than VV-dTK/PE. We therefore propose further optimization of the VV-dTK/EE backbone to improve tumor selectivity.

### 3.3 Deleting F4L in VV- dTK/EE improves tumor selectivity and safety

**Figure 2:**
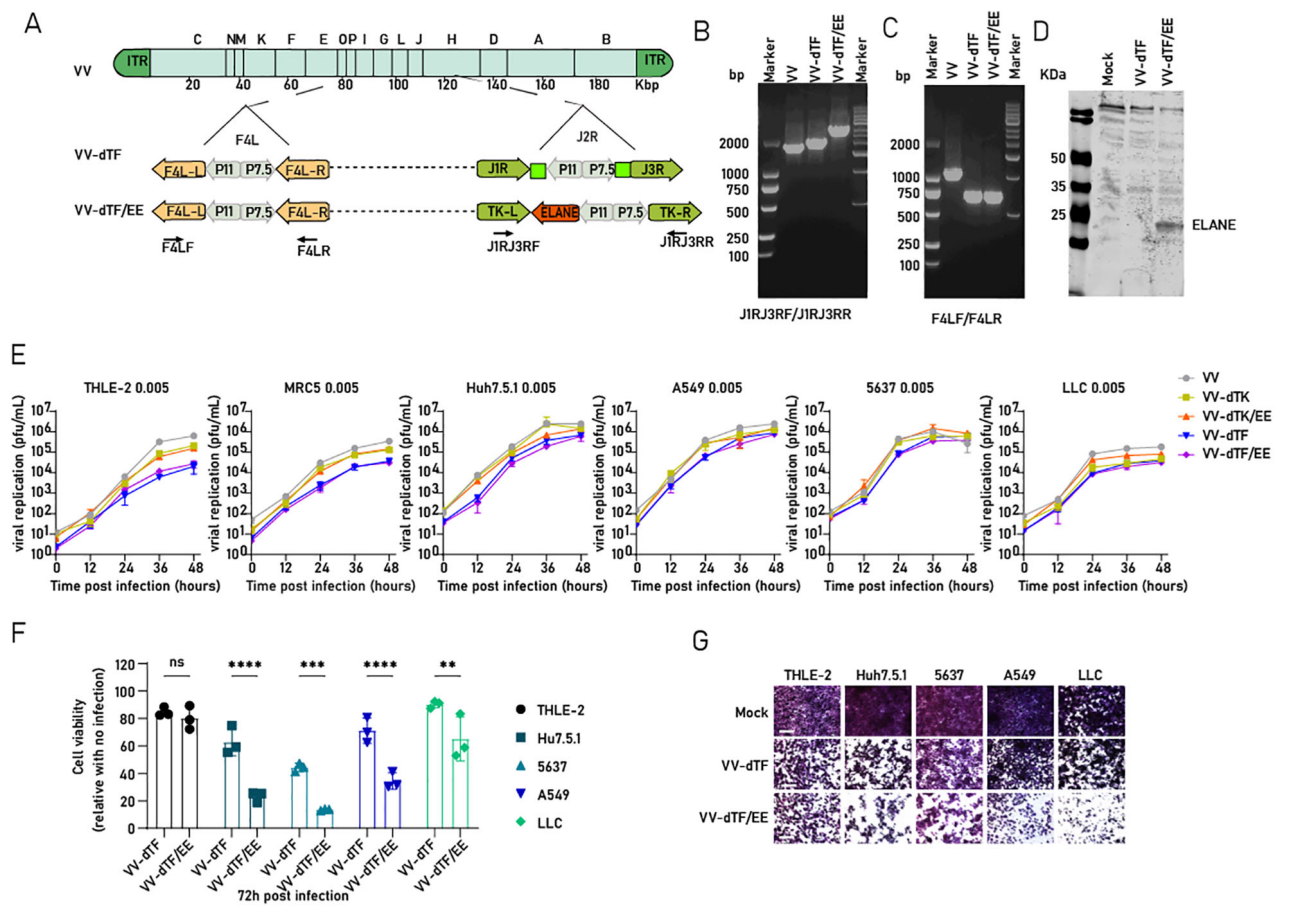
Deletion of F4L improves safety and tumor selectivity of VV-dTK/EE. **(A)** Schematic representation of VV-dTF/EE and locations of PCR primers. **(B, C)** PCR analysis of recombinant viruses to confirm the substitution of ELANE for the TK region **(B)** and deletion of F4L **(C, D)** Expression of ELANE protein in cells infected by VV-dTF or VV-dTF/EE at MOI = 0.5 for 24 h. **(E)** Cells were infected with the indicated VACV at MOI = 0.005. Viruses were harvested 0, 12, 24, 36, or 48 h after infection, viral titers were determined by plaque assays in Vero cells. **(F)** Cell viability was measured using the CCK8 assay, 72 h after infection with the indicated VACV. Data were calculated using the cell number of no-infection control as 100. The statistical analysis was estimated by one-way ANOVA with Tukey’s correction. All data are presented as mean values ± SD. *p < 0.05; **p < 0.01; ***p < 0.001; ****p < 0.0001; ns, not significant. **(G)** Cells were infected with the indicated VACV at MOI = 1. 24 h after infection, cells were fixed with 4% paraformaldehyde for 15 minutes at room temperature, stained with crystal violet. Microscopy images of plaque morphology. Scale bar, 100 μm.

To increase the safety of VV-dTK/EE, we deleted the *F4L* gene which encodes the ribonucleotide reductase small subunit (RRM2), to further attenuate this recombinant VACV (**Figure 2A**). *F4L*/*J2R* deletion and ELANE expression were detected by PCR and Western blot (**Figures 2B–D**), this new recombinant VACV was named VV-dTF/EE. Both VV-dTF and VV-dTF/EE were poorly infectious in normal human liver cell THLE-2, produced a titer of 1x10^4^ PFU/mL at 48 hours post infection (MOI = 0.005), which is about 100-fold lower than wild-type VACV Tiantan strain and 10-fold lower than both VV-dTK and VV-dTK/EE (**Figure 2E**). In human embryonic lung fibroblast MRC5 cells, the replication titers of VV-dTF and VV-dTF/EE were both at 1*10^4^ PFU/mL at 48 hours post infection (MOI = 0.005) which is about 30-fold lower than wild-type VACV and 10-fold lower than both VV-dTK and VV-dTK/EE (**Figure 2E**). In contrast, the replication of VV-dTF/EE in human cancer cells Huh7.5.1, 5637 and A549 was only moderately reduced compared with VACV (about 10 folds lower) or VV-dTK/EE (3 to 5 folds lower), and was able to produce viruses of more than 1x10^5^ PFU/mL at 48 hours post infection (**Figure 2E**). Although VACV infection was detectable in mouse LLC tumor cells at ~10^4^ PFU/mL, its overall replication capacity in murine cells was markedly lower that in human cell lines (**Figure 2E**).

When cell viability was measured with the CCK8 assay at 72 hpi at MOI = 0.05, more than 80% of normal hepatocytes THLE-2 survived infection of either VV-dTF or VV-dTF/EE (**Figure 2F**). In contrast, human tumor cells Huh7.5.1, 5637 and A549 exhibited cell viability of 63.1%, 44.4%, and 71.1% after VV-dTF infection, as opposed to 23.2%, 13.6%, and 34.5% cell viability after infection of VV-dTF/EE (**Figure 2F**). In addition, microscopic analysis with high MOI = 1 at 24 hpi revealed more severe death of tumor cells with VV-dTF/EE infection compared with VV-dTF (**Figure 2G**).

**Figure 3:**
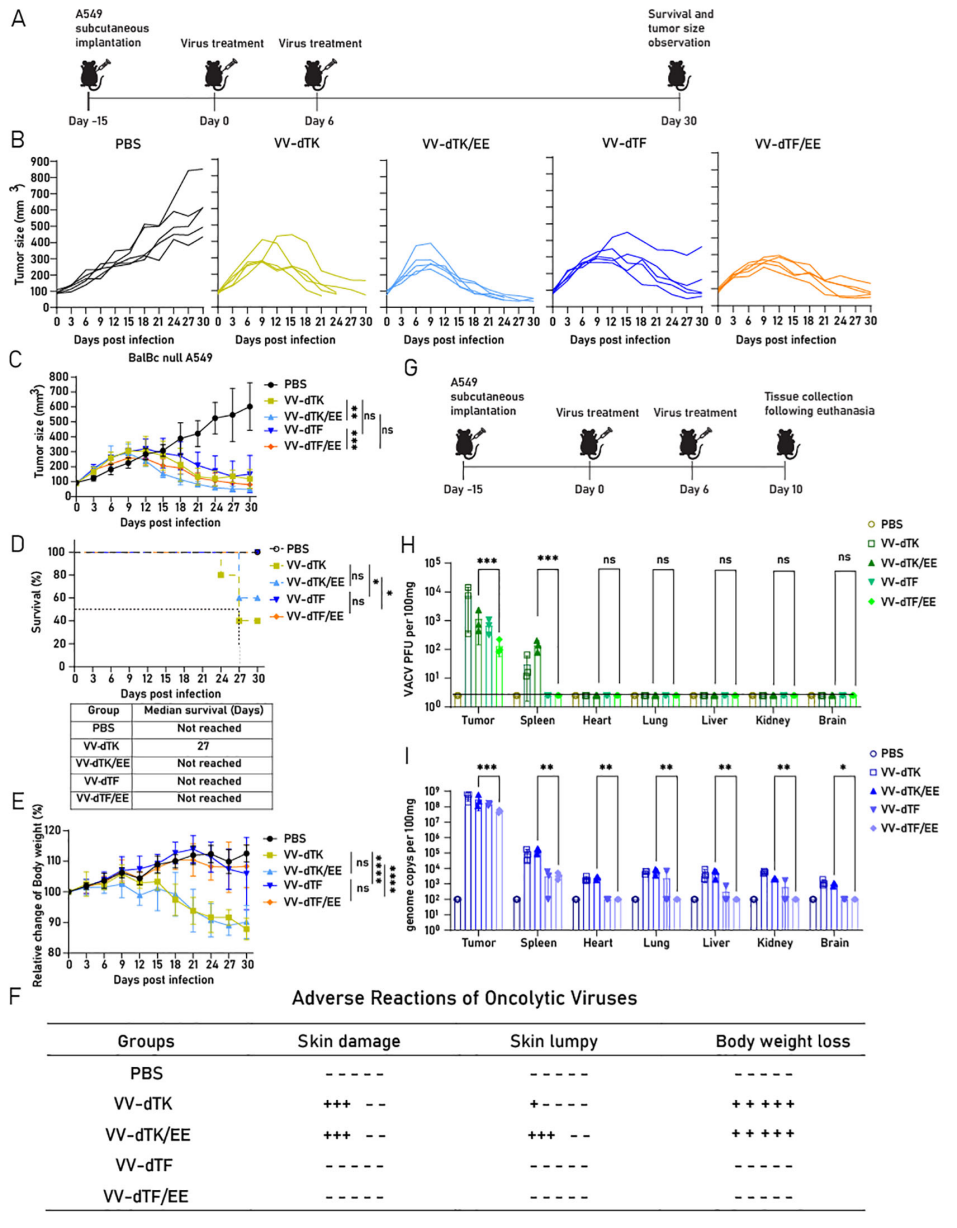
Specific tumor killing and safety of VV-dTF/EE in A549 Balb/c nude mice. **(A)** Schedule of A549 tumor implantation and treatment. BALB/c nude mice harboring subcutaneous A549 tumors were randomized and injected twice (day 0 and 6) with an intratumoral dose of 5x10^5^ PFU of VV-dTK or VV-dTK/EE. PBS was used as a control. **(B–D)** Tumor size in individual animals and **(B)** mean tumor size after treatments **(C)**, overall survival **(D)** and body weight changes **(E)** are plotted. The median survival rate is shown in the table below **(D)**. Each experimental group has 5 mice. Statistical analysis was performed with two-way ANOVA of **(C, E)** with Tukey’s correction. Log-rank test was performed for the data in **(D)**. All data are presented as mean values ± SD. **(F)** Adverse reactions of A549 subcutaneous tumor-bearing mice. **(G)** Schedule of VACV infection and tissue collection. **(H)** Virus titers in different tissues on day 4 after infection. Organs were harvested and homogenized. Virus titers were determined by plaque assays in Vero cells. Each group has 3 mice. **(I)** Copy number of VACV genomes was determined by PCR. Statistical analysis was performed with one-way ANOVA with Tukey’s correction of **(H, I)**. Data are presented as mean values ± SD. *p < 0.05; **p < 0.01; ***p < 0.001; ****p < 0.0001; ns, not significant.

We next used NSCLC A549 Balb/c null mice model to assess the safety and biodistribution of VV-dTF/EE. After the tumors became palpable (~150cm^3^), two doses of each virus at 5x10^5^ PFU or PBS control were administered by intratumoral injection with a 6-day interval (**Figure 3A**). All tested recombinant VACVs reduced the tumor volume (**Figures 3B, C**) with ELANE-expressing VACVs showing stronger inhibition than control VACV vector either in the context of *TK* or *TK/F4L* deletion (**Figures 3B, C**). Under this treatment condition, there was no significant difference between VV-dTK vs VV-dTF or VV-dTK/EE vs VV-dTF/EE (**Figures 3B, C**). Both VV-dTF and VV-dTF/EE significantly increased mouse survival compared to the VV-dTK and VV-dTK/EE (**Figure 3D**), whereas no death occurred in the VV-dTF or VV-dTF/EE groups during the observation period (**Figure 3D**). In addition, unlike VV-dTK and VV-dTK/EE that caused loss of mouse body weight, VV-dTF and VV-dTF/EE infections showed no significant effect on mouse body weight compared with the PBS control group (**Figure 3E**). Both the VV-dTK and VV-dTK/EE groups exhibited marked weight loss during treatment, and there was no significant difference of weight loss between VV-dTK and VV-dTK/EE group, or between VV-dTF and VV-dTF/EE group (**Figure 3E**). Further investigation showed that as opposed to VV-dTK and VV-dTK/EE that caused lethargy, progressive cachexia, and abscess development along with reduced survival (**Figure 3F**; **Supplementary Figure 3**), VV-dTF and VV-dTF/EE infections did not show these morbidity and mortality.

Lastly, we performed plaque assay and viral genome PCR to measure viral distribution in heart, liver, spleen, lung, kidney, brain and tumor that were collected from A549 tumor-bearing mice 4 days after recombinant VACV treatment (**Figures 3G–I**). Due to the sensitivity limit of the plaque assay, only homogenized supernatants from tumor and spleen tissues gave visible plaques. The results showed that VV-dTK produced the highest titers in tumors, reaching 10^4^ PFU/100 mg, whereas VV-dTK/EE showed lower titers of 10³ PFU/100 mg. Both VV-dTK/EE and VV-dTF/EE produced virus titers approximately 10-fold lower than VV-dTK and VV-dTK/EE in tumor tissues. In splenic tissues, only homogenates from the VV-dTK and VV-dTK/EE groups generated detectable plaques, no plaques were observed in the VV-dTF and VV-dTF/EE groups (**Figure 3H**). Viral genomes in the collected tissues were quantified using VACV E9L-targeted qPCR. All recombinant VACV strains demonstrated efficient replication in tumor tissues, with VV-dTF/EE exhibiting 10-fold lower viral genome copies than VV-dTK/EE. In spleens, VV-dTF/EE also had significantly lower viral genome copies than VV-dTK/EE. Notably, VV-dTF/EE was nearly undetectable in heart, lung, liver, kidney and brain, whereas VV-dTK/EE had measurable replication across all examined tissues (**Figure 3I**).

Collectively, these data suggest that VV-dTF/EE maintains its selective tumor-killing efficacy with significantly reduces distribution and replication in non-tumor tissues.

### 3.4 VV-dTF/EE infection induces apoptosis and ICD in tumor cells

**Figure 4:**
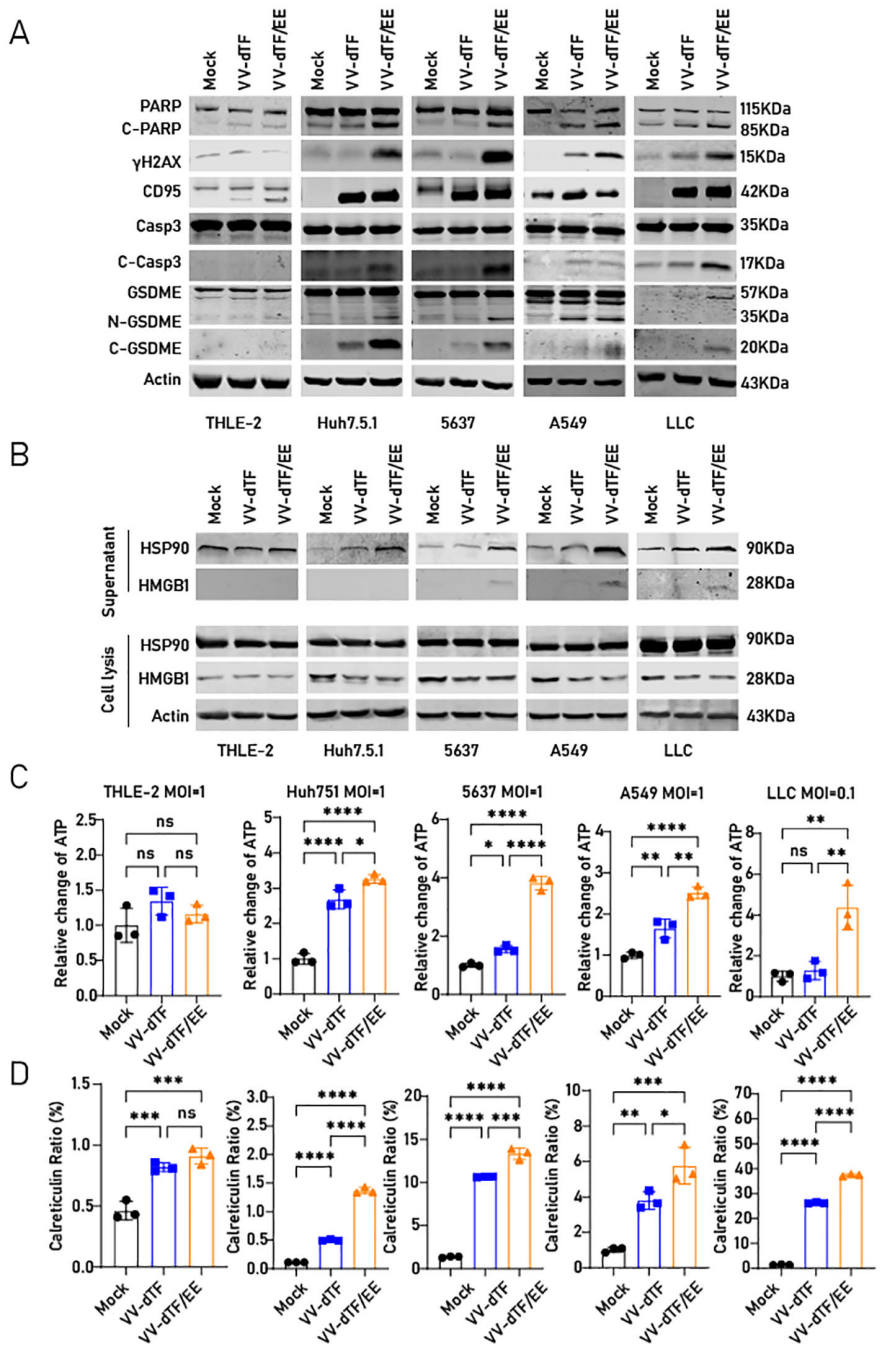
VV-dTF/EE induces apoptosis and ICD in tumor cells. **(A)** Western blots to detect markers of apoptosis (PARP and Caspase 3 cleavage), DNA damage (γH2AX), and pyroptosis (GSDME) in cells infected with VV-dTF or VV-dTF/EE at MOI = 1 for 24 h. **(B)** ICD makers HMGB1 and HSP90 were detected in cell lysates and culture supernatants by Western blots 24 h post infection by VV-dTF or VV-dTF/EE at MOI = 1. **(C)** Release of ATP to culture supernatants after infection. Cells were infected with the indicated viruses at MOI = 1. Levels of ATP was measured. **(D)** Cells expressing CRT on their surfaces after VACV infections. Statistical analysis was performed with one-way ANOVA with Tukey’s correction for the data shown in **(C, D)**. All data are presented as mean values ± SD. *p < 0.05; **p < 0.01; ***p < 0.001; ****p < 0.0001; ns, not significant.

To further characterize the oncolytic property of the VV-dTF/EE virus, we assessed its ability to trigger multiple programmed cell death pathways in tumor cells, including apoptosis, pyroptosis, and ICD. The results showed that as opposed to VV-dTF that moderately enhanced the cleavage of apoptosis markers caspase-3 and PARP, and the levels of DNA damage in A549 and LLC tumor cells, and slightly increased pyroptosis in Huh7.5.1 and 5637 tumor cells (**Figure 4A**), VV-TF/EE significantly stimulated apoptosis, DNA damage response and cleavage of GSDME in all cancer cells examined (**Figure 4A**). In addition, the histone H1 isoform rapidly translocated from the nucleus to the cytoplasm (**Supplementary Figure 4**) where it interacts with CD95 death domain and contributes to tumor cell killing.

Moreover, infection with VV-dTF/EE significantly enhanced the secretion of the immunogenic cell death (ICD) markers HSP90, HMGB1and ATP from infected cancer cells compared with VV-dTF (**Figures 4B, C**), whereas non-cancer cells show no significant changes upon infection by either VV-dTF or VV-dTF/EE (**Figures 4B, C**). Likewise, VV-dTF/EE infection of cancer cells caused more translocation of calreticulin (CRT) from the cytoplasm onto the cell surface than VV-dTF (**Figure 4D**), although did not show marked effect on the THLE-2 non-cancer cells. These results suggest that VV-dTF/EE infection induces more apoptosis and ICD in cancer cells compared with VV-dTF/EE, which could cause the release of tumor-associated antigens (TAAs) and the launch of antitumor immune response (36).

### 3.5 VV-dTF/EE infection enhances the infiltration of CD8^+^ T cells

**Figure 5:**
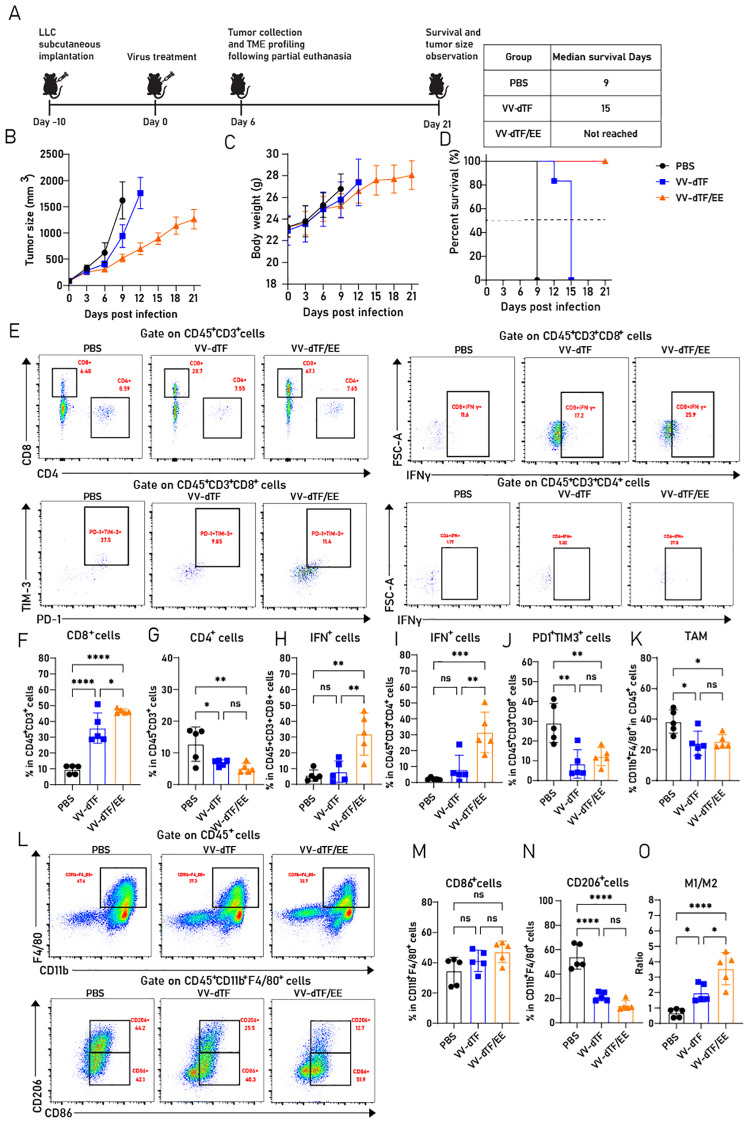
VV-dTF/EE improves anti-tumor efficacy by increasing CD8+ T cell infiltration and relieving immunosuppression. **(A)** Schedule of LLC tumor implantation and VACV treatment. C57BL/6 mice harboring subcutaneous LLC tumors were randomized and injected with an intratumoral dose of 1x10^6^ PFU VV-dTF or VV-dTF/EE. PBS was used as a control. **(B)** mean tumor size after treatments, body weight changes **(C, D)** overall survival are plotted (n=6). The median survival rate is shown in the table above the **(D, E)** Representative flow cytometry scatterplots for F to J, each panel represents an individual sample. **(G–K)** Percentages of CD8+ **(G)**, CD4+ **(H)**, CD8+IFNγ+ **(I)**, CD4+IFNγ+ **(J)**, and PD1+TIM3+ **(K)** T cells in LLC tumor tissues. **(L)** Levels of tumor associated macrophages (TAMs) in non-infected and infected LLC tumor tissues. **(M)** Representative flow cytometry scatterplot of TAM [in **(L)**] and M1 (in N), M2 [in **(O)**] macrophages. **(N, O)** Levels of M1 **(N)** and M2 **(O)** macrophages in non-infected and infected LLC tumor tissues. **(P)** Ratios of M1 to M2 macrophages in tumor tissues. All data were derived from individual samples; no concatenation was applied. Statistical analysis was performed with one-way ANOVA with Tukey’s correction for the data shown in **(F–K)** and **(M–O)**. All data are presented as mean values ± SD. *p < 0.05; **p < 0.01; ***p < 0.001; ****p < 0.0001; ns, not significant.

Next, we measured the effect of VV-dTF/EE on host anti-tumor immune responses using a syngeneic mouse lung cancer model which was established with C57BL/6 mice bearing sub-cutaneous LLC tumors. Mice received intratumor injections of 1x10^6^ PFU of recombinant VACVs (**Figure 5A**). Compared to the PBS control group, virus-treated tumors grew much slower, and the anti-tumor effect of VV-dTF/EE was more robust than VV-dTF (**Figure 5B** and S5A). No evident weight loss and mortality were observed in any treatment group (**Figure 5C**). It took significant longer for the VV-dTF/EE-treated tumors to reach 2500 mm^3^ in volume (maximal tumor volume allowed by ethics) than VV-dTF (**Figure 5B**; **Supplementary Figure 5A**). The survival rate of VV-dTF/EE-treated mice was significantly higher than the VV-dTF and the PBS groups (**Figure 5D**). When ICD markers (HSP90 and HMGB1) were detected 6 dpi in tumor tissues by immunohistochemistry (**Supplementary Figure 5B**), much higher levels were observed with the VV-dTF/EE group (**Supplementary Figure 5B**).

Given the key role of T cells in antitumor immunity, especially cytotoxic CD8^+^ T cells (37–39), we investigated T cell infiltration in tumor tissues following VACV infection (**Supplementary Figure 6A**). The results of immunofluorescence profiling showed that VV-dTF/EE infection increased the number of CD8^+^T cells in tumor tissues more than VV-dTF (**Supplementary Figure 5B**). Further analysis of T cell profiles in tumor tissues by flow cytometry at 6 dpi showed increase of CD8^+^ T cell infiltration in VACV-treated tumor tissues, and this increase was the greatest with VV-dTF/EE (**Figures 5E, F**). In contrast, modest reduction of CD4^+^ T cell populations was observed with VACV treatment (**Figures 5E, G**). Notably, both IFNγ-positive cytotoxic CD8^+^ T cells and CD4^+^ T cells (Th1) underwent considerable expansion in the VV-dTF/EE group (**Figures 5E, H, I**), which indicate a robust activation of the tumor immune microenvironment by VV-dTF/EE.

Since the immunosuppressive tumor microenvironment (TME) is known to play an important role in tumor progression and drug resistance (39, 40), we thus examined the effect of VV-dTF/EE treatment on the presence of immunosuppressive cells in tumor tissues, including regulator T cells (Tregs), exhausted T cells and tumor-associated macrophages (TAMs) (**Supplementary Figure 6B**). The results showed that both VV-dTF and VV-dTF/EE reduced the number of exhausted CD8^+^T cells which were scored as PD1^+^TIM3^+^CD8^+^ T cells (**Figures 5E, J**). The number of Treg cells (CD25^+^Foxp3^+^CD4^+^) decreased slightly without reaching statistical significance (**Supplementary Figure 5C**). VV-dTF and VV-dTF/EE significantly increased the ratios of CD8^+^ T cells to Tregs (**Supplementary Figure 5D**). These results suggest that the effect on T cell immunosuppressive activity mainly depends on VV-dTF, ELANE expression does not make significant contribution.

TAMs participate in the establishment of the tumor microenvironment which promotes tumor growth, invasion, metastasis, and drug resistance (41–43). Both VV-dTF and VV-dTF/EE significantly reduced TAM infiltration in tumor tissues (**Figures 5K, L**). TAM subtype analysis showed that the reduction mainly occurred to M2-polarized TAMs (CD206^+^F4/80^+^CD11b^+^) (**Figures 5L, N**). Notably, ELANE expression resulted in a more pronounced increase in the ratio of M1/M2 TAMs (**Figure 5O**). Recent studies have also reported that ELANE can promote M1 polarization (44); however, it remains unclear whether this effect is mediated directly through ELANE’s protease activity or indirectly via ELANE−induced immunogenic cell death and pyroptosis. The underlying molecular mechanisms warrant further investigation.

Taken together, these data suggest that VV-dTF/EE infection alters the tumor microenvironment by increasing immune cell infiltration, activating tumor-infiltrated effector T cells, and reducing exhausted CD8^+^ T cells and TAM M2 polarization.

## 4 Discussion

In this study, we engineered recombinant VACV to express ELANE, a tumor-selective protease secreted by neutrophils, through inserting this gene into the *J2R* locus, and deleted viral genes *F4L*to further generated a recombinant oncolytic virus of VV-dTF/EE with high tumor selectivity and excellent safety. Compared with the conventional replication-deficient VACV MVA vectors, that typically require repeated high-dose administrations (45, 46), our recombinant VV-dTF/EE maintains robust replication capacity in tumor cells and mice tumor models, thus demonstrates significant antitumor efficacy with a two-dose regimen.

Most oncolytic vaccinia viruses developed to date carry mutations inactivating the *J2R* gene. This gene encodes viral thymidine kinase, a key enzyme in the salvage pathway of nucleotide biosynthesis. Deletion of *J2R* has been demonstrated to attenuate viral virulence while preserving viral replication capacity in dividing cells. However, Johann Foloppe et al. (2019) found that the level of intracellular TK does not significantly affect the replication of △*J2R* VACV (47). Our results also observed VV-dTK and VV-dTK/EE treatment-related adverse effects such as body weight loss, skin coarsening, and mortality (**Figures 3E, F**). On this basis, we further disrupted the *F4L* gene, which encodes the ribonucleotide reductase small subunit (RRM2), to improve the tumor selectivity and safety profile of the recombinant oncolytic virus.

Cancer cells frequently undergo metabolic reprogramming due to aberrant oncogenic signaling, shifting toward anabolic metabolism to supply macromolecules and deoxynucleoside triphosphates (dNTPs) required for cell division (48) and generally exhibit a higher proportion of cells in S-phase compared to normal tissues (49). This high nucleotide metabolic environment is conducive to the selective replication of VV-dTF/EE in tumors. As illustrated in **Figure 4**, compared to the VV-dTK/EE (which lacks *J2R*), VV-dTF/EE maintained robust antitumor activity while enhancing survival and preserving body weight in tumor-bearing mice. Furthermore, associated adverse effects—including skin lesion formation—were substantially mitigated. Biodistribution studies in mice confirmed that VV-dTF/EE displays enhanced tumor-specific tropism.

ELANE has been demonstrated tumor-specific cytotoxic activity (4). It cleaves CD95 to generate a death domain that recognizes tumor-specific histone components, thereby selectively inducing cell death in malignancies (4). This intrinsic mechanism of ELANE further bolsters the tumor selectivity and safety profile of the VV-dTF/EE construct. More interesting, we observed that VACV infection upregulates CD95 levels in multiple cancer cell lines. Since CD95 is a substrate of ELANE, this high level of CD95 enhances the anti-tumor efficacy of VV-dTF/EE.

Furthermore, a very distinct type of cell death within the virus plaques was observed when comparing VV-dTF with VV-dTF/EE infection, which is likely attributed to distinct cell death pathways triggered by VV-dTF/EE. Indeed, we detected a marked increase in the cleaved, activated form of GSDME, an effector molecule of pyroptosis. ELANE has also been shown to cleave GSDMD into active GSDMD‐NT which induced pyroptosis (50). Pyroptosis is a highly inflammatory form of programmed cell death, is able to alleviate immunosuppression and promote systemic immune responses in solid tumors (51). In subsequent immunogenic cell death (ICD) assays, we consistently observed that VV-dTF/EE provoked a robust release of key ICD markers, including HSP90, HMGB1, and ATP, along with the translocation of calreticulin (CRT), both *in vitro* (**Figures 4B–D**) and in murine tumor tissues (**Supplementary Figure 5B**).

In the LLC syngeneic tumor model, VV-dTF/EE delayed tumor growth and significantly increased mouse survival. It does so by modulating the tumor immune microenvironment, such as enhancing CD8^+^ T cell infiltration, diminishing CD4^+^ T cells to shift toward cytotoxic immune responses. Although VV-dTF alone can exert some of these effects, expression of ELANE from VV-dTF/EE significantly augments these outcomes, and markedly increases the proportion of IFNγ-expressing CD8^+^ effector T cells. Furthermore, VV-dTF/EE treatment also alters the immunosuppressive tumor microenvironment (TME). This is characterized by reduced T cell exhaustion, although no significant difference was observed between VV-dTF and VV-dTF/EE, suggesting that lack of T cell exhaustion is primarily mediated by VV-dTF. Notably, ELANE expression significantly inhibits M2-polarized macrophage infiltration in the TME, indicating its specific role in modulating myeloid cell populations.

## 5 Study limitations

Several limitations of this study should be acknowledged. First, this study did not include *in situ* models or additional syngeneic tumor models, the efficacy of VV-dTF/EE and immune infiltration patterns may differ in orthotopic settings. Second, the lack of analyses of draining lymph nodes, dendritic cells, and antigen peptide stimulated immune infiltration limits our understanding of T cell priming, cytokine production, and antigen specificity. Third, although ELANE is human-derived, its substrates and interacting proteins in mice may not completely reflect those in humans, potentially affecting mechanistic interpretation. Fourth, this study did not evaluate the efficacy of VV-dTF/EE in combination with standard immune checkpoint inhibitors (anti-PD-1/CTLA-4), which warrants future investigation. These limitations should be considered when interpreting our findings and highlight directions for future work.

## 6 Conclusion

In this study, we have developed a genetically modified VACV expressing ELANE, which selectively replicates in and kill tumor cell while modifying the tumor immune microenvironment to elicit immunogenic cell death. Collectively, our study presents a new and promising strategy of cancer therapy by combining oncolytic virus VACV and tumor killing gene ELANE. Future studies will use orthotopic models and additional more syngeneic tumor models to analyze the anti-tumor responses, including tumor rechallenge to assess immunological memory, and test combination with immune checkpoint inhibitors, advancing clinical translation.

## Data Availability

The original contributions presented in the study are included in the article/**Supplementary Material**. Further inquiries can be directed to the corresponding author.

## Supplementary material

Supplementary Figure 1 VV-dTK/EE specifically kills tumor cells. **(A)** PCR analysis of recombinant VACVs to confirm the substitution of ELANE or PPE for the TK region. **(B)** Expression of ELANE and PPE proteins in cells 24 h after infection by the recombinant VACVs at MOI = 0.5. **(C)** Vero cells were infected with recombinant virus MOI = 1. After 24 h, the supernatant was collected and ultrafiltered through a 50kDa cutoff membrane (4 °C, 4000×g, 30 min). The virus-free filtrate was assessed for ELANE activity. This filtrate was then added to 5637 cancer cells for 24 h, followed by Western blot detection of CD95 cleavage. **(D)** Programmed cell death after VACV infections. Western blots were performed with cell lysates 24 h after infection with the indicated VACV at MOI = 1.

Supplementary Figure 2 VV-dTK/EE induces ICD in cancer cells. **(A)** Western blots to detect ICD markers HSP90 and HMGB1 in cell lysis and culture supernatant infected with the indicated VACV recombinants. **(B)** Release of ATP into culture supernatants after VACV infection. Cells were infected with the indicated viruses at 24 h post infection. The luminescent detection kit was used to measure ATP levels. **(C)** Percentages of cells expressing CRT on their surface at 24 h post infection. Results are the average of three independent infections. Statistical analysis was performed with one-way ANOVA with Tukey’s correction. All data are presented as mean values ± SD. *p < 0.05; **p < 0.01; ***p < 0.001; ****p < 0.0001; ns, not significant.

Supplementary Figure 3 Representative image of adverse reactions with the indicated VACV injection related with **Figure 3F**. The damaged skin areas are marked with red arrows.

Supplementary Figure 4 VV-dTF/EE induces translocation of H1 isoforms from the nucleus to the cytoplasm. Detection of H1.2 (green) in Huh7.5.1 and 5637 cells by immunofluorescence staining. Blue, DAPI staining of nuclei. Scale bar, 20 μm.

Supplementary Figure 5 ICD and Treg induced of VV-TF/EE in LLC tumor. **(A)** Tumor size in individual animals related with **Figure 5B**. **(B)** Schedule of VACV treatment and tissue collection. Representative images of immunohistochemistry staining for ICD markers (HSP90 and HMGB1) and immunofluorescence staining for CD8^+^ T cells in LLC tumor tissues. Scale bar, 200 μm. Quantification of HSP90 and HMGB1 expression by using Image J (n=3). The statistical analysis was estimated by one-way ANOVA with Tukey’s correction. All data are presented as mean values ± SD. *p < 0.05; **p < 0.01; ***p < 0.001; ****p < 0.0001; ns, not significant. **(C)** Levels of CD25^+^FoxP3^+^CD4^+^ regulatory T cells in LLC tumor tissues 6 days after VACV infection. **(D, E)** Ratios of CD4^+^T cells **(D)** or CD8^+^ cells **(E)** to Treg cells in tumor tissues (n=5). Statistical analysis was performed with one-way ANOVA with Tukey’s correction. Data are presented as mean values ± SD. *p < 0.05; ns, not significant.

Supplementary Figure 6 Gating strategy of flow cytometry to locate T cells **(A)** and TAMs **(B)**.
