# Supplementary material for: J2R/F4L deleted oncolytic vaccinia virus synergizes with tumor specific killer (ELANE) promotes antitumor immunity with superior safety
Source: Front Immunol. 2026 Jul 21;17:1861346. doi: 10.3389/fimmu.2026.1861346 (PMC13433746; doi:10.3389/fimmu.2026.1861346)
Supplement: Supplementary file 1 [file DataSheet1.docx]

**Supplementary Information**

**Fig S1. VV-dTK/EE specifically kills tumor cells.**

(A) PCR analysis of recombinant VACVs to confirm the substitution of ELANE or PPE for the TK region. (B) Expression of ELANE and PPE proteins in cells 24 h after infection by the recombinant VACVs at MOI=0.5. (C)Vero cells were infected with recombinant virus $\mathrm{MOI}=1$. After 24 h, the supernatant was collected and ultrafiltered through a 50kDa cutoff membrane (4 °C, 4000×g, 30 min). The virus‑free filtrate was assessed for ELANE activity. This filtrate was then added to 5637 cancer cells for 24 h, followed by Western blot detection of CD95 cleavage. (D) Programmed cell death after VACV infections. Western blots were performed with cell lysates 24 h after infection with the indicated VACV at MOI=1.

**Fig S2. VV-dTK/EE induces ICD in cancer cells.**

(A) Western blots to detect ICD markers HSP90 and HMGB1 in cell lysis and culture supernatant infected with the indicated VACV recombinants. (B) Release of ATP into culture supernatants after VACV infection. Cells were infected with the indicated viruses at 24 h post infection. The luminescent detection kit was used to measure ATP levels. (C) Percentages of cells expressing CRT on their surface at 24 h post infection. Results are the average of three independent infections. Statistical analysis was performed with one-way ANOVA with Tukey’s correction. All data are presented as mean values ±SD. *p < 0.05; **p < 0.01; ***p < 0.001; ****p < 0.0001; ns, not significant.

**Fig S3. Representative image of adverse reactions with the indicated VACV injection related with Figure 3F.**

The damaged skin areas are marked with red arrows.

**Fig S4. VV-dTF/EE induces translocation of H1 isoforms from the nucleus to the cytoplasm**

Detection of H1.2 (green) in Huh7.5.1 and 5637 cells by immunofluorescence staining. Blue, DAPI staining of nuclei. Scale bar, 20 μm.

**Fig S5. ICD and Treg induced of VV-TF/EE in LLC tumor.**

(A) Tumor size in individual animals related with figure 5B. (B) Schedule of VACV treatment and tissue collection. Representative images of immunohistochemistry staining for ICD markers (HSP90 and HMGB1) and immunofluorescence staining for CD8^+^ T cells in LLC tumor tissues. Scale bar, 200 μm. Quantification of HSP90 and HMGB1 expression by using Image J (n=3). The statistical analysis was estimated by one-way ANOVA with Tukey’s correction. All data are presented as mean values ± SD. *p < 0.05; **p < 0.01; ***p < 0.001; ****p < 0.0001; ns, not significant. (C) Levels of CD25^+^FoxP3^+^CD4^+^ regulatory T cells in LLC tumor tissues 6 days after VACV infection. (D and E) Ratios of CD4^+^T cells (D) or CD8^+^ cells (E) to Treg cells in tumor tissues (n=5). Statistical analysis was performed with one-way ANOVA with Tukey’s correction. Data are presented as mean values ±SD. *p < 0.05; ns, not significant.

**Fig S6. Gating strategy of flow cytometry to locate T cells (A) and TAMs (B).**

**Table1 Antibody information**

| Antibody name | Target | Fluorophore | Source | Identifier | Dilution ratio |
| --- | --- | --- | --- | --- | --- |
| PARP Antibody | PARP | - | CST | 9542S | 1:1000 |
| Caspase3 Antibody | Caspase3 | - | CST | 9662S | 1:1000 |
| HMGB1 (D3E5) Rabbit Monoclonal Antibody | HMGB1 | - | CST | 6893S | 1:1000 |
| HSP90 Polyclonal antibody | HSP90 | - | Proteintech | 13171-1-AP | 1:1000 |
| DFNA5/GSDME Polyclonal antibody | GSDME | - | Proteintech | 13075-1-AP | 1:1000 |
| Fas/CD95 Polyclonal antibody | CD95 | - | Proteintech | 13098-1-AP | 1:1000 |
| Anti-β-Actin (ACTB) Antibody | Actin | - | Sigma | A1978 | 1:1000 |
| Anti-phospho-Histone H2A.X (Ser139) Antibody, clone JBW301 | γH2AX | - | Sigma | 05-636 | 1:1000 |
| Neutrophil elastase Antibody [N2C3] | ELANE | - | GeneTeX | GTX113175 | 1:1000 |
| allophycocyanin (APC)-labeled anti-CRT polyclonal antibody | calreticulin | APC | Proteintech | 10427-2-AP | 1:1000 |
| BV786 Rat Anti-Mouse CD45 | Mouse CD45 | BV786 | BD Bioscience | 564225 | 1:200 |
| PE-Cy™7 Hamster Anti-Mouse CD3e | Mouse CD3 | PE-Cy™7 | BD Bioscience | 552774 | 1:200 |
| RB705 Rat Anti-Mouse CD4 | Mouse CD4 | RB705 | BD Bioscience | 570258 | 1:200 |
| BV510 Rat Anti-Mouse CD8a | Mouse CD8 | BV510 | BD Bioscience | 563068 | 1:200 |
| BB515 Rat Anti-Mouse CD25 | Mouse CD25 | BB515 | BD Bioscience | 564424 | 1:100 |
| PE Rat Anti-Mouse IFN-γ | Mouse IFN-γ | PE | BD Bioscience | 554412 | 1:100 |
| PE-CF594 Hamster Anti-Mouse CD279 (PD-1) | Mouse CD279 (PD-1) | PE-CF594 | BD Bioscience | 562523 | 1:100 |
| RB705 Rat Anti- Mouse CD11b | Mouse CD11b | RB705 | BD Bioscience | 570292 | 1:200 |
| BV421 Rat Anti-Mouse F4/80 | Mouse F4/80 | BV421 | BD Bioscience | 565411 | 1:100 |
| BV650 Rat Anti-Mouse CD86 | Mouse CD86 | BV650 | BD Bioscience | 564200 | 1:100 |
| PE Rat Anti-Mouse CD206 | Mouse CD206 | PE | BD Bioscience | 568273 | 1:100 |
| BV711™ anti-mouse CD366 (Tim-3) | mouse CD366 (Tim-3) | BV711™ | Biolegend | 119727 | 1:50 |
| BV421™ anti-mouse FOXP3 | mouse FOXP3 | BV421™ | Biolegend | 126419 | 1:50 |
|  |  |  |  |  |  |
